# Evaluation of Maternal-Fetal Medicine Fellowship Program Websites

**DOI:** 10.7759/cureus.58527

**Published:** 2024-04-18

**Authors:** Nathanael N Hoskins, Hannah Daley, Marco A Cunicelli

**Affiliations:** 1 Obstetrics and Gynecology, Reading Hospital, West Reading, USA; 2 Obstetrics and Gynecology, Drexel University College of Medicine, West Reading, USA; 3 Family Medicine, Lancaster General Hospital, Lancaster, USA

**Keywords:** maternal fetal medicine fellowship, graduate medical education, virtual interview, fellowship website, diversity equity and inclusion (dei), ob/gyn fellowship, obstetrics and gynecology (ob/gyn), fellowship, maternal fetal medicine

## Abstract

Introduction

Due to the COVID-19 pandemic, the American Association of Medical Colleges (AAMC) recommended that all interviews for residencies and fellowships be conducted in a virtual format. As of March 2024, the Society of Maternal-Fetal Medicine (SMFM) continues to request that all fellowship interviews occur virtually. Without in-person interviews, prospective Maternal-Fetal Medicine (MFM) fellowship applicants must largely rely on program websites to gain insight into each program’s offerings, culture, and application requirements. The purpose of this study was to evaluate the content of American College of Graduate Medical Education (ACGME)-accredited Maternal-Fetal Medicine (MFM) fellowship program websites and assess if regional differences exist among website content.

Methods

All ACGME-accredited MFM fellowship program websites were assessed for 21 defined criteria as of March 2024 and further compared by geographic regions (Midwest, Northeast, South, and West). Analyses were completed using chi-squared univariate tests, with a p < 0.05.

Results

Of the 108 accredited MFM fellowship programs, 106 programs had a dedicated website (98.15%). Most MFM programs (over 80%) included contact information (102/106), program director name (98/106), faculty names (95/106), application requirements (92/106), current fellow names (91/106), and the program coordinator name (89/106) on their website. Less programs (less than 30%) included diversity, equity, inclusion (DEI) content (28/106), interview dates (28/106), and current fellow research projects or publications (27/106). Western programs were less likely to include the program coordinator's name (12/18 (67%), p = 0.046), but more likely to include DEI content (10/18 (56%), p = 0.005). Northeastern programs were less likely to include their application requirements (24/32 (75%), p = 0.049) and less likely to include pictures of their current fellows (20/32 (63%), p = 0.045). Southern programs were more likely to include the yearly rotation schedule (19/32 (59%), p = 0.040). Midwestern programs were more likely to include information on fellowship benefits or salary (15/24 (63%), p = 0.046).

Conclusion

This study demonstrated that the content available on MFM fellowship websites varies greatly between programs and geographic regions. Efforts should be made by MFM training institutions to enhance website DEI content, curriculum information, recent fellow publications, and information on program alumni. A detailed and well-structured website may help applicants compare individual programs more equitably in the age of virtual interviewing.

## Introduction

Due to the COVID-19 pandemic, the American Association of Medical Colleges (AAMC) recommended that all interviews for residencies and fellowships be conducted in a virtual format [1]. As advances have been made in the prevention and treatment of COVID-19, many restrictions have been lifted. However, these restrictions caused an indefinite shift in the manner in which interviews for residency and fellowship are performed. Virtual interviews eliminate travel costs and minimize time away from work. As a result, it has been suggested that online interviews likely decrease potential bias towards applicants able to travel to an in-person interview and are more equitable [2]. Therefore, as of March 2024, the Society of Maternal-Fetal Medicine (SMFM) continues to request that all fellowship interviews occur virtually [3].

Without in-person interviews, prospective Maternal-Fetal Medicine (MFM) fellowship applicants must largely rely on program websites to gain insight into each program’s offerings, culture, and application requirements. Therefore, website content may impact which programs prospective fellows choose to apply for and the applicant’s initial impression of each program.

Several studies have analyzed the content of various residency and fellowship program websites [4-13]. Other studies have demonstrated the importance of fellowship websites when applying to and ranking programs [14-16]. To our knowledge, there has not been a study analyzing the content available on MFM fellowship websites. The purpose of this study is to assess the specific information on MFM fellowship websites and determine if there is variation in content across different geographical regions of the United States.

## Materials and methods

The Accreditation Council for Graduate Medical Education (ACGME) is the organization responsible for accrediting all graduate medical training programs for physicians in the United States, including internships, residencies, and fellowships. This organization maintains a list of every accredited training program. A list of all ACGME-accredited MFM fellowships was obtained from their website (www.acgme.org) on March 1, 2024. To evaluate the accessibility of each fellowship program’s website, a Google search (www.google.com) was performed. The search phrase included each program’s name followed by “MFM fellowship.” Each program’s website was accessed by an initial reviewer and the evaluation criteria, discussed below, were logged in a Microsoft Excel spreadsheet. The spreadsheet and each program’s website were then re-examined by a second reviewer to ensure the accuracy of data entry. The websites were accessed for the initial data entry between March 1, 2024, and March 5, 2024, by the primary reviewer. The validation process occurred over the following three weeks by the secondary reviewer.

Each website was evaluated to assess for the presence or absence of 21 pre-determined criteria (Table 1). The criteria were selected based on previous research evaluating website content for residency and fellowship programs and applicant survey data [4-14]. To be included in the study, the criteria had to either be directly available on the MFM fellowship website or directly linked to the fellowship website. For organizational purposes, the criteria were divided into five categories as listed in Table 1.

**Table 1:** Criteria evaluated on MFM Fellowship websites MFM: Maternal-Fetal Medicine

Evaluation Category	Evaluation Criteria
General program information	Clinical site(s) or facility description
	Contact information (phone number or email)
	Program mission/aims
	Department-specific diversity, equity, and inclusion (DEI) policy or content
	Salary or benefits
Application details	Application requirements
	Application deadline
	Interview dates
Academic information	Didactics
	Procedural/surgical exposure
	Yearly rotation schedule
Research	Faculty publications
	Current fellow publications
Program personnel	Program director name
	Program coordinator/administrator name
	Faculty names
	Current fellow names
	Current fellow education
	Current fellow pictures
	Alumni names
	Alumni current positions

To allow for further examination of regional trends, each program was also assigned to a geographic region. These were assigned using the regions as defined on the United States Census Bureau website (www.census.gov) and included Midwest, Northeast, South, and West. For statistical analysis, a chi-squared univariate analysis was used with a p < 0.05 considered statistically significant.

## Results

As of March 1, 2024, there were 108 ACGME-accredited MFM fellowships (Table 2). Of those, there were 106 MFM fellowship programs with a dedicated website (98.15%). The two programs without a website were marked as “newly accredited” on the ACGME website. Of the 106 programs included in the data analysis, 24 were in the Midwest, 32 were in the Northeast, 32 were in the South, and 18 were in the West.

**Table 2:** All ACGME-accredited MFM fellowship programs ^1^Programs marked as "newly accredited" on the ACGME website ACGME, American College of Graduate Medical Education; MFM, Maternal-Fetal Medicine; UCLA, University of California, Los Angeles; UMass, University of Massachusetts; B-JH, Barnes-Jewish Hospital; SLCH, St. Louis Children's Hospital; NYU, New York University; SUNY, The State University of New York; UPMC, University of Pittsburgh Medical Center; TJUH, Thomas Jefferson University Hospital; SOM, School of Medicine

ACGME-Accredited MFM Fellowship Programs			
University of Alabama Medical Center Program	University of Arizona College of Medicine-Phoenix Program	University of Arizona College of Medicine-Tucson Program	University of Arkansas for Medical Sciences (UAMS) College of Medicine Program
Cedars-Sinai Medical Center Program	Stanford Health Care-Sponsored Stanford University Program	University of California (Irvine) Program	University of California (San Francisco) Program
University of Southern California/Los Angeles General Medical Center (USC/LA General) Program	UCLA David Geffen School of Medicine/UCLA Medical Center Program	University of California (San Diego) Medical Center Program	Arrowhead Regional Medical Center Program
University of California Davis Health Program	University of Colorado Program	Yale-New Haven Medical Center Program	University of Connecticut Program
MedStar Health/Washington Hospital Center Program	University of South Florida Morsani Program	University of Florida Program	University of Miami/Jackson Health System Program
Emory University School of Medicine Program	Medical College of Georgia Program	University of Hawaii Program	University of Chicago Program
McGaw Medical Center of Northwestern University Program	Loyola University Medical Center Program	University of Illinois College of Medicine at Chicago Program	Indiana University School of Medicine Program
University of Iowa Hospitals and Clinics Program	University of Kansas School of Medicine Program	University of Kentucky College of Medicine Program	Ochsner Clinic Foundation Program
Johns Hopkins University Program	University of Maryland Program	Beth Israel Deaconess Medical Center Program	Mass General Brigham/Massachusetts General Hospital Program
Mass General Brigham/Brigham and Women's Hospital Program	Tufts Medical Center Program	UMass Chan Medical School Program	UMass Chan - Baystate Program^1^
University of Michigan Program	Corewell Health East Beaumont (Royal Oak) Program	Detroit Medical Center/Wayne State University Program	Henry Ford Health/Henry Ford Hospital Program
Corewell Health – Grand Rapids/Michigan State University Program	Mayo Clinic College of Medicine and Science (Rochester) Program	University of Minnesota Program	University of Mississippi Medical Center Program
SSM Health/Saint Louis University School of Medicine Program	University of Missouri-Kansas City School of Medicine Program	Washington University/B-JH/SLCH Consortium Program	Rutgers Health/New Jersey Medical School Program
Rutgers Health/Robert Wood Johnson Medical School Program	University of New Mexico School of Medicine Program	Icahn School of Medicine at Mount Sinai/Mount Sinai Hospital Program	Icahn School of Medicine at Mount Sinai/West Program
Stony Brook Medicine/University Hospital Program	University of Rochester Program	Albany Medical Center Program	New York Presbyterian Hospital (Columbia Campus) Program
Zucker School of Medicine at Hofstra/Northwell Program	Montefiore Medical Center/Albert Einstein College of Medicine Program	New York Presbyterian Hospital (Cornell Campus) Program	NYU Grossman School of Medicine Program
Maimonides Medical Center Program	NYU Grossman Long Island School of Medicine Program	Zucker School of Medicine at Hofstra/Northwell at South Shore University Hospital Program	SUNY Upstate Medical University Program
University of North Carolina Hospitals Program	Mountain Area Health Education Center Program	Wake Forest University Baptist Medical Center Program	Duke University Hospital Program
Ohio State University/Mt Carmel Hospital Program	The MetroHealth System/Case Western Reserve University Program	University of Cincinnati Medical Center/College of Medicine Program	Cleveland Clinic Foundation Program
Case Western Reserve University/University Hospitals Cleveland Medical Center Program	University of Oklahoma Health Sciences Center Program	Oregon Health & Science University (OHSU Health) Program	Penn State Milton S Hershey Medical Center Program
UPMC Medical Education Program	Sidney Kimmel Medical College at Thomas Jefferson University/TJUH Program	University of Pennsylvania Health System Program	Geisinger Health System Program
UPMC Medical Education (Harrisburg) Program	Lehigh Valley Health Network Program	Women and Infants Hospital of Rhode Island/Brown University Program	Medical University of South Carolina Program
Prisma Health/University of South Carolina SOM Greenville (Greenville) Program	University of Tennessee Program	Vanderbilt University Medical Center Program	University of Tennessee Medical Center at Knoxville Program
Baylor College of Medicine Program	University of Texas Medical Branch Hospitals Program	University of Texas Health Science Center San Antonio Joe and Teresa Lozano Long School of Medicine Program	University of Texas Southwestern Medical Center Program
University of Texas Health Science Center at Houston (Memorial Hermann Hospital) Program	University of Texas at Austin Dell Medical School Program	University of Utah Health Program	University of Vermont Medical Center Program
University of Virginia Medical Center Program	Inova Fairfax Medical Campus Program^1^	Eastern Virginia Medical School Program	Virginia Commonwealth University Health System Program
University of Washington Program	Madigan Army Medical Center Program	University of Wisconsin Hospitals and Clinics Program	Medical College of Wisconsin Affiliated Hospitals Program

**Figure 1:**
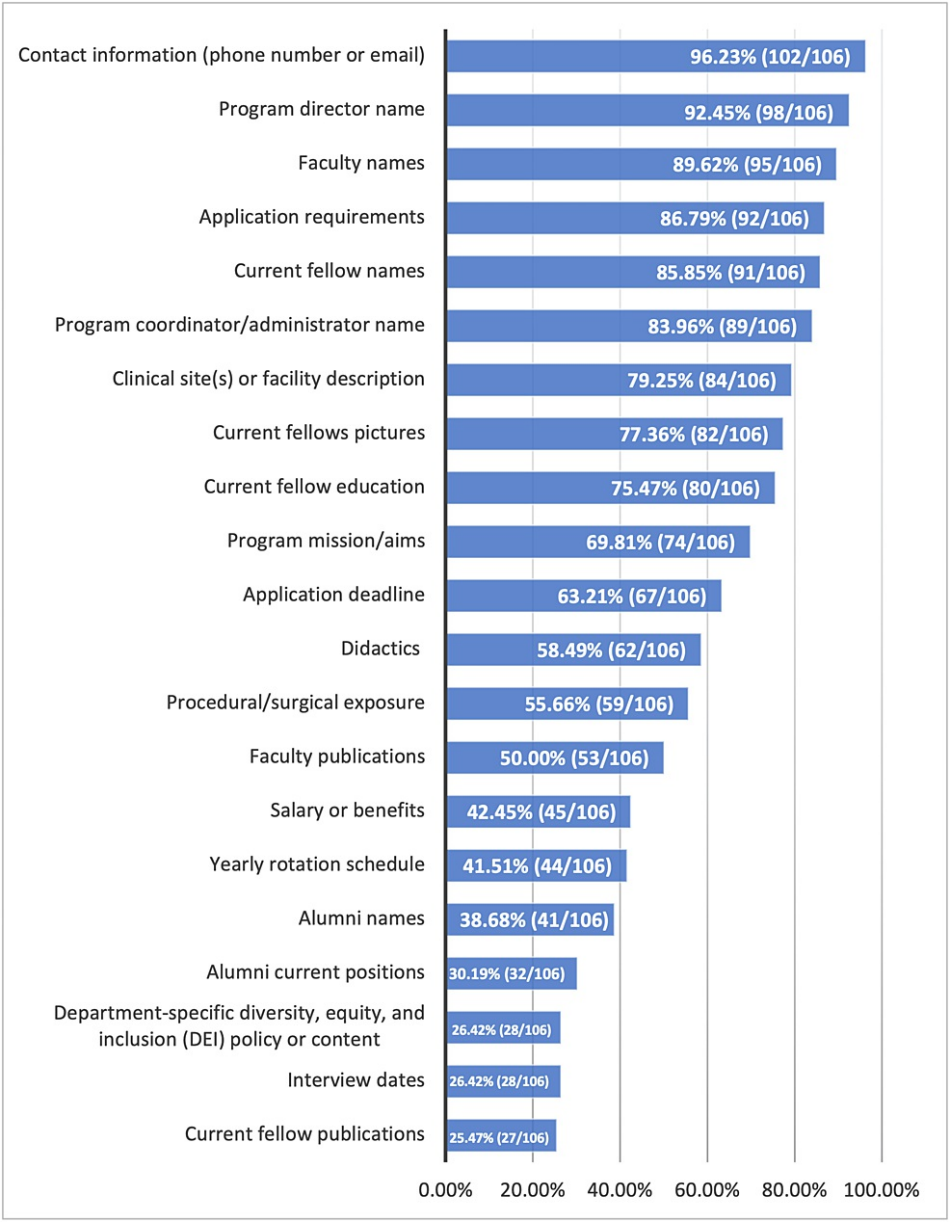
Summary of MFM fellowship program website content Percentage of MFM Fellowship program websites that include each of the pre-determined criteria MFM, Maternal-Fetal Medicine

A majority of MFM programs (over 80%) included contact information (102/106), program director name (98/106), faculty names (95/106), application requirements (92/106), current fellow names (91/106), and the program coordinator/administrator name (89/106) on their website. Less than 30% of the websites included information regarding DEI content (28/106), interview dates (28/106), and current fellow research projects or publications (27/106). A detailed summary of the findings is included in Figure 1.

There were multiple significant findings when comparing regional differences in MFM program websites. Western programs were less likely to include the program coordinator/administrator name (12/18 (67%), p = 0.046) and Northeastern programs were less likely to include their application requirements (24/32 (75%), p = 0.049). Overall, 77% (82/106) of programs included pictures of their current fellows. However, Northeastern programs were less likely to include pictures of their current fellows (20/32 (63%), p = 0.045). Southern programs were more likely to include the yearly rotation schedule (19/32 (59%), p = 0.040). Midwestern programs were more likely to include information on fellowship benefits or salary (15/24 (63%), p = 0.046). Overall, 26% (28/106) of MFM programs included DEI information on their website. However, Western program websites were more likely to include a program-specific DEI policy (10/18 (56%), p = 0.005). There were no other statistically significant regional differences among the other criteria evaluated.

## Discussion

This study aimed to evaluate the content of maternal-fetal medicine (MFM) fellowship program websites and assess regional trends in content. The results indicate that the majority of ACGME-accredited MFM fellowships have a dedicated website. This is an encouraging finding, as it suggests that MFM programs recognize the importance of providing accessible information to potential applicants, especially in the age of virtual interviewing. Virtual interviews prevent applicants from visiting programs in person to tour the facilities and gauge the climate of the program. Fellowship program websites provide programs the opportunity to help bridge this gap and showcase their culture and unique offerings.

Across all regions, most websites provided program contact information and application requirements. Providing an email address or phone number offers applicants a means to reach out with any questions or concerns that arise during the application cycle. Additionally, having clear application requirements helps to ensure that prospective fellows understand individual program expectations. Most websites also provided the names of the program director and faculty members. This may help applicants gain insight into program leadership and faculty expertise, and identify potential mentors within the program.

A 2016 study surveyed interventional radiology fellowship applicants to determine what content they valued on program websites. This study found didactic information, facility information, and rotation schedules to be the most valued content by applicants [16]. This content was only found in 58% (59/106), 79% (84/106), and 42% (44/106) of websites in our study, respectively (Figure 1). While one cannot conclude this desired content can be broadly applied to all specialties, it does likely highlight areas for improvement on many MFM program websites. There were several other content deficiencies noted among program websites. Many websites lacked content on current fellow research projects or publications and interview dates. Information on current fellow research may be helpful for applicants looking for programs with ongoing studies in a particular area of interest. This is particularly important for residents applying to MFM, as the ACGME requires at least 12 months of protected research time throughout the fellowship [17]. Additionally, less than 30% of analyzed websites included information on DEI. This is not a new finding; a study published in 2023 found that over 80% of MFM fellowship programs showed minimal elements promoting DEI on their website [14]. They also noted a trend toward Western MFM programs, including more DEI content on their website; however, this lacked statistical significance [14]. In contrast, our study did find a statistically significant proportion of Western programs included DEI content or policies compared to other regions, although still only 56%. The ACGME program requirements now emphasize the importance of systematic recruitment and retention of a diverse and inclusive workforce of residents and fellows [17]. Incorporating DEI statements or initiatives into their websites may help MFM programs attract more diverse applicants and foster an inclusive learning environment.

Several additional regional factors were significant. Western programs were less likely to include the name of the program coordinator or administrator on their website compared to other regions. This discrepancy may raise questions about communication accessibility within these programs. Midwestern programs were more likely to include detailed information regarding fellowship benefits or salary on their websites. This transparency can be advantageous for prospective applicants for whom benefit offerings may carry increased weight in their decision-making.

This study has several limitations. Data were initially collected over five days and were validated over several weeks. It is possible that websites may have been updated during this period and that some websites may have been updated between data collection and publication. For example, interview dates are often added later in the application cycle, as they may be dependent upon faculty availability. Additionally, each website was evaluated only for the presence or absence of specific content, not the detail. For example, some websites included only a sentence on their DEI policy, whereas others had a dedicated DEI section. Finally, the criteria chosen for assessment were based on prior studies from other medical specialties [4-14]. While these criteria can likely be extrapolated to prospective MFM fellows, there may be other website content that could be more important to these applicants.

## Conclusions

This study provides insight into the current content of MFM fellowship program websites and highlights areas for improvement. It also demonstrated that the content of MFM fellowship websites varies greatly between programs and geographic regions. Efforts should be made by MFM training institutions to enhance website DEI content, curriculum information, recent fellow publications, and information on program alumni. These improvements would likely help applicants to compare individual programs more equitably. Future studies could explore whether changes implemented based on these findings lead to increased applicant satisfaction, and ultimately influence recruitment outcomes in MFM fellowships.
